# Higher Risk of Gestational Diabetes in Women With a Greater Variety of Sleep Disturbances

**DOI:** 10.1155/jp/4604151

**Published:** 2026-05-03

**Authors:** Joanna Laurila, Johanna Masar, Marjo Tuppurainen, Soili M. Lehto, Sari Hantunen, Leea Keski-Nisula

**Affiliations:** ^1^ Institute of Clinical Medicine, School of Medicine, University of Eastern Finland, Kuopio, Finland, uef.fi; ^2^ Department of Obstetrics and Gynecology, Kuopio University Hospital, Kuopio, Finland, psshp.fi; ^3^ Institute of Clinical Medicine, University of Oslo, Oslo, Norway, uio.no; ^4^ R&D Department, Division of Mental Health Services, Akershus University Hospital, Lørenskog, Norway, ahus.no; ^5^ Department of Psychiatry, University of Helsinki and Helsinki University Hospital, Helsinki, Finland, helsinki.fi; ^6^ Institute of Public Health and Clinical Nutrition, University of Eastern Finland, Kuopio, Finland, uef.fi

**Keywords:** gestational diabetes mellitus, glucose, pregnancy, sleep disturbance, weight gain

## Abstract

**Objective:**

Sleep disturbances and gestational diabetes mellitus (GDM) are both common during pregnancy. However, it remains unclear whether sleep disturbances are independent risk factors for the development of GDM. This study examined subjectively reported sleep disturbances during the second trimester and their association with GDM and related maternal factors.

**Methods:**

A total of 5055 pregnant women from the Finnish Kuopio Birth Cohort completed an online questionnaire assessing sleep disturbances (sleep quality, sleep duration, weekly snoring, difficulties in falling asleep, daytime sleepiness, and nighttime or early morning awakenings) during the second trimester. Among the 4976 women with completed follow‐up to delivery, GDM was detected in 23.1% (*N* = 1151) according to the Finnish National Current Care Guidelines. Adjusted logistic regression analyses (controlling for maternal age, body mass index [BMI], smoking, physical activity, and gestational weight gain) were used to examine the associations between sleep disturbances and GDM.

**Results:**

Difficulty falling asleep (aOR 1.35; 95% CI 1.05–1.72), nighttime or early morning awakenings (aOR 1.16; 95% CI 1.00–1.34), and frequent (aOR 1.21; 95% CI 1.02–1.44) and daily daytime sleepiness (aOR 1.52; 95% CI 1.14–2.03) remained significant risk factors for GDM after adjusting for covariates. Furthermore, the risk of GDM increased in a linear manner with the number of reported sleep disturbances (aORs 1.07–1.41). A higher first‐trimester BMI was the strongest predictor of experiencing multiple sleep disturbances. Among women with GDM, sleep problems were more frequent in those requiring pharmacological treatment compared with those managed with lifestyle interventions.

**Conclusion:**

Sleep disturbances are strongly associated with the development of GDM. Screening for and managing sleep disturbances should be considered part of routine maternity care; improving sleep quality may support healthier overall lifestyle habits and better glucose balance during pregnancy.

## 1. Introduction

Gestational diabetes mellitus (GDM), defined as impaired glucose metabolism first detected during pregnancy and resulting in hyperglycemia of variable severity [1], has become a major public health concern among pregnant women worldwide. Its prevalence varies depending on the region and diagnostic criteria applied. According to the International Diabetes Federation, approximately 17% of pregnancies in 2021 were affected by hyperglycemia, of which 80.3% of these cases were attributed to GDM [2]. In Finland, nearly one in five pregnant women is diagnosed with GDM, and the prevalence has increased by approximately 10% over the past decade, primarily due to the obesity epidemic [3]. Maternal GDM is strongly associated with the development of fetal macrosomia and labor dystocia, as well as early neonatal hypoglycemia, hyperbilirubinemia, and respiratory distress syndrome. It is also strongly linked to an increased risk of long‐term metabolic problems not only in the mother but also in the offspring, including obesity, hypertension, and dyslipidemia [4–7].

The risk of developing GDM is multifactorial and associated with various maternal and prenatal factors, including advanced maternal age, prepregnancy overweight or obesity, cigarette smoking during pregnancy, a sedentary lifestyle, poor diet, a previous history of GDM, carrying a male fetus, multiple pregnancy, a strong familial history of diabetes, genetic predisposition, ethnicity, polycystic ovary syndrome, and psychosocial factors [4]. Given that GDM is linked to a range of adverse maternal and neonatal outcomes, as well as long‐term effects on the offspring [5–7], it is essential to identify modifiable risk factors for hyperglycemia during pregnancy.

Pregnancy brings various anatomic and hormonal changes that can lead to psychological distress and physical discomfort, both of which can adversely affect maternal sleep [8]. In a study of 2427 pregnant women in the United States, the majority reported poor sleep quality (76%), insufficient nighttime sleep (38%), daytime sleepiness (49%), symptoms of insomnia (57%), sleep‐disordered breathing (19%), and restless legs syndrome (24%) [9]. All participants experienced frequent nighttime awakenings, with 83% attributing them to frequent urination and 79% to difficulty finding a comfortable sleeping position. Most (78%) women also reported taking daytime naps. Sleep disturbances were most prevalent during the third trimester, when women experienced later bedtimes, more frequent and prolonged nighttime awakenings, shorter nighttime sleep, and reduced total sleep duration [9]. Moreover, sleep disturbances have been associated with maternal complications and adverse fetal outcomes, including preeclampsia, gestational hypertension, preterm birth, cesarean delivery, stillbirth, and large‐for‐gestational‐age infants. According to a recent meta‐analysis involving nearly 60 million pregnant women, these risks were particularly pronounced among older and overweight women [10].

Sleep disturbances may impair glucose metabolism and increase the risk of diabetes. Previous studies have identified sleep disturbances as independent risk factors for hyperglycemia and type 2 diabetes in nonpregnant populations [11–13], and they may also contribute to the development of hyperglycemia during pregnancy [14]. However, many earlier studies examining the association between sleep disturbances and the risk of GDM have been conducted in Asian populations, where the proportion of overweight or obese pregnant women has been relatively low [15–19]. As a result, the influence of pregestational weight or gestational weight gain on the relationship between sleep disturbances and GDM may have been underestimated. Rawal et al. [20] investigated the associations between GDM, sleep duration, and daytime napping in a racially diverse cohort of pregnant women in the United States, with a high proportion of overweight and obese participants (43.6%). Interestingly, a significant association between GDM and sleep duration in the second trimester was observed only among nonobese women.

Both sleep disturbances and GDM are common during pregnancy and appear to be associated; however, the independence of this association and the role of potential modifying factors remain unclear. Therefore, we examined whether sleep disturbances were associated with GDM during the second trimester of pregnancy in a sample of 4976 women from the Kuopio Birth Cohort (KuBiCo) study. We also investigated whether these associations could be explained by demographic or maternal factors. Second, we aimed to characterize sleep quality in a large birth‐cohort population. We hypothesized that sleep disturbances are strongly associated with specific maternal factors, such as advanced maternal age, obesity, and smoking, and that the association between sleep disturbances and GDM would be attenuated when these risk factors are included in the analysis.

## 2. Materials and Methods

### 2.1. Study Population

Participants were recruited into the KuBiCo study during their first prenatal clinic visit (gestational weeks [GWS] 6–9) by healthcare personnel or midwives. KuBiCo, initiated in 2012, is a population‐based pregnancy cohort study aimed at evaluating prenatal factors that influence the health of mothers and children living in the Finnish region of Northern Savo [21].

All participants provided electronic informed consent and were expected to deliver at Kuopio University Hospital (KUH) [22]. At the time of this study in January 2026, a total of 5055 pregnant women had completed an online questionnaire assessing sleep during the second trimester. Women who did not give birth at KUH or had not yet delivered at the time of data analysis were excluded from the analysis (*N* = 79) evaluating the association between gestational diabetes and sleep problems. Thus, the final sample used to evaluate the outcome variables included 4976 women.

### 2.2. Internet‐Based Questionnaire

The sleep questionnaire was incorporated into the KuBiCo database in September 2014. It included six questions assessing sleep quality during the second trimester of pregnancy (GWS 13–28), and participants completed the questionnaire during the third trimester.

For statistical analysis, we categorized sleep disturbances into the following six groups: (1) worsened sleep quality compared to prepregnancy (response options: “no change or better” vs. “yes”); (2) snoring, defined as self‐reported snoring during pregnancy (ranging from “no or do not know” to “yes, 1–2 nights a week” and “yes, ≥ 3 nights a week”); (3) abnormal nightly sleep duration, defined as sleeping ≤ 6 h or ≥ 9 h per night compared to 7–8 h; (4) difficulty falling asleep, reported as occurring “sometimes” or “often or almost every night”; (5) nighttime or early morning awakenings, reported as occurring “sometimes” or “often or almost every night”; and (6) excessive daytime sleepiness, defined as feeling sleepy during the day “frequently, at least weekly” or “almost daily.”

In addition, we calculated a sleep disturbance burden score by summing the number of reported disturbances across the six categories. Each disturbance contributed one point to the total score, resulting in a possible range from 0 (no disturbances) to 6 (all six disturbances present during the second trimester).

### 2.3. Blood Glucose Measurement and Diagnosis of GDM

GDM was diagnosed using a 75‐g, 2‐h oral glucose tolerance test (OGTT), typically performed in Finland between GWS 24 and 28. Diagnostic thresholds followed the Finnish Current Care Guidelines: fasting glucose ≥ 5.3 mmol/L, 1‐h postload glucose ≥ 10.0 mmol/L, and 2‐h postload glucose ≥ 8.6 mmol/L [3]. Approximately 70%–80% of pregnant women in Finland undergo OGTT during pregnancy [3]. Women who do not undergo OGTT are generally considered to be at low risk for GDM or have preexisting diabetes. Low risk is defined by the following criteria: a pregnant woman is (1) aged < 25 years, nulliparous with normal weight (body mass index [BMI] 18.5–24.9 kg/m^2^) and no family history of type 2 diabetes or (2) aged < 40 years, primi‐ or multiparous with BMI < 25kg/m^2^ and no history of GDM or macrosomia in previous pregnancies or no family history of type 2 diabetes. Women at high risk for GDM typically undergo OGTT earlier, between GWS 12 and 16. High risk is defined by the presence of at least one of the following: (1) history of GDM in previous pregnancies; (2) BMI ≥ 30 kg/m^2^ and/or waist circumference > 90 cm at the beginning of pregnancy; (3) glucosuria in early pregnancy; (4) first‐degree relative with type 2 diabetes; (5) use of oral corticosteroids; or (6) diagnosis of nonalcoholic fatty liver disease. If the initial OGTT result is normal in a high‐risk woman, the test is usually repeated between GWS 24 and 28 [3]. Women with preexisting diabetes who did not undergo OGTT during pregnancy were included in the group without a GDM diagnosis. In this study, 3765 women (75.7%) underwent OGTT during pregnancy, reflecting national figures [3].

### 2.4. Covariates

Information on years of education, employment status during pregnancy, marital status, and physical activity was obtained from questionnaires completed during pregnancy. Additionally, maternal caffeine intake was estimated from 1924 pregnancies using a 160‐item food frequency questionnaire completed during the first trimester (GWS 2–12) as part of the KuBiCo study [23]. Data on maternal age, height, weight change during pregnancy, parity, cigarette smoking, pregestational diabetes and hypertension, use of corticosteroids during pregnancy, and infertility treatments prior to the current pregnancy were recorded in medical records during pregnancy and retrieved at the time of the study.

First‐trimester BMI was calculated using weight (kg) divided by height squared (m^2^), based on measurements taken at the first maternity clinic visit (typically between GWS 8 and 12). BMI was categorized according to the World Health Organization classification: underweight and normal weight (BMI < 25.0 kg/m^2^), overweight (BMI ≥ 25.0 and < 30.0 kg/m^2^), obese (BMI ≥ 30.0 and < 35.0 kg/m^2^ and ≥ 35.0 and < 40.0 kg/m^2^), and severely obese (BMI ≥ 40.0 kg/m^2^). Due to the small number of underweight women in the study population (*n* = 32; 0.8%), underweight (BMI < 18.0 kg/m^2^) and normal weight (BMI ≥ 18.0 and < 25.0 kg/m^2^) categories were combined. Gestational weight gain was calculated as the difference between maternal weight at delivery and weight measured during the first trimester.

### 2.5. Statistical Analysis

The prevalence of sleep disturbances was presented as percentages.In the univariate analyses, categorical variables were evaluated using Pearson’s chi‐square test, and continuous variables were analyzed using analysis of variance (ANOVA).

Explanatory variables for poor sleep quality included maternal age, parity, first‐trimester BMI (kg/m^2^), smoking, daily caffeine intake during the first trimester (mg/day), physical activity, relationship status, education, employment, infertility treatment prior to the current pregnancy, pregestational diabetes, pregestational hypertension, use of oral corticosteroids during pregnancy, and number of fetuses. Additional explanatory variables for the occurrence of sleep disturbances included GDM status (yes/no), timing of abnormal OGTT (< 28 or ≥ 28 GWS), GDM severity (lifestyle vs. pharmaceutical treatment), and gestational weight gain. Women with pregestational diabetes (*n* = 36; 0.7%) were included in the analyses examining associations between GDM and sleep disturbances.

Logistic regression analyses were conducted to examine the associations between GDM and various sleep disturbances in multivariable models and to estimate odds ratios (ORs) for GDM. Both crude and adjusted odds ratios (aORs) with 95% confidence intervals (95% CIs) were calculated, controlling for potential confounders. Covariates were selected based on their established relevance to GDM in previous studies, including maternal age, BMI, smoking, physical activity [24], and gestational weight gain [25, 26]. All covariates were entered simultaneously into the logistic regression models and treated as categorical variables. A subgroup analysis was performed using multivariable logistic regression for women who underwent OGTT (*n* = 3765), using the same covariates. Maternal parity was not associated with GDM in the univariate analysis (data not shown) and was therefore excluded from the regression models. All selected covariates were significantly associated with GDM in the logistic regression analysis.

All statistical tests were two‐tailed, and a *p* value of < 0.05 was considered statistically significant. Analyses were conducted using IBM SPSS Statistics Version 27 (Armonk, New York, United States).

## 3. Results

### 3.1. Quality of Sleep

Table 1 presents the self‐reported sleep disturbances in the study population. Among the 5055 pregnant women, 52.8% (*n* = 2670) reported that their subjective sleep quality had deteriorated during the second trimester compared to prepregnancy. Weekly snoring was reported by 24.1% (*n* = 1216), while 11.0% (*n* = 556) reported short (≤ 6 h) and 17.9% (*n* = 905) long (≥ 9 h) sleep duration. Frequent difficulty falling asleep was reported by 8.5% (*n* = 432), frequent nighttime or early morning awakenings by 35.7% (*n* = 1806), and frequent daytime sleepiness by 25.0% (*n* = 1263).

**Table 1:** Quality of sleep during the second trimester among 5055 pregnant women in the Kuopio Birth Cohort study.

Sleep variables	*N*	%
Sleep quality compared to nonpregnancy		
No change or better	2385	47.2
Worse	2670	52.8
Snoring		
No or do not know	3839	75.9
Yes, 1–2 nights a week	596	11.8
Yes, ≥ 3 nights a week	620	12.3
Sleep duration (h)		
≤ 6	556	11.0
7–8	3594	71.1
≥ 9	905	17.9
Difficulty in falling asleep		
No	2426	48.0
Sometimes	2197	43.5
Often or almost every night	432	8.5
Nighttime or early morning awakenings		
No	759	15.0
Sometimes	2490	49.3
Often or almost every night	1806	35.7
Daytime sleepiness		
No or cannot define	3792	75.0
Frequently, at least weekly	998	19.7
Almost daily	265	5.2
Total	5055	100

### 3.2. Demographics and Sleep Characteristics

Table 2 presents the demographic characteristics of the study population. Half of the participants (52.1%) were nulliparous, and the majority were aged between 25 and 35 years (72.1%), were married or in an equivalent relationship (95.6%), were employed (78.8%), and had a minimum of 16 years of education (65.5%). Most women (59.8%) had a first‐trimester BMI of < 25 kg/m^2^, although a substantial proportion were overweight (23.9%) or obese (16.4%). A total of 36 women (0.7%) had pregestational diabetes, and 49 (1.0%) had pregestational hypertension. Additionally, 71 women (1.4%) were carrying twins.

**Table 2:** Maternal characteristics and poor sleep quality in the second trimester among 5055 pregnant women.

	Total	Worse sleep quality compared to nonpregnancy		Weekly snoring		Sleep duration ≤ 6 or ≥ 9 h		Often difficulties in falling asleep		Often nighttime or early morning awakenings		Weekly daytime sleepiness		Sleep disturbance burden^b^	
	*n* (%)	*n* (%)	*p* value	*n* (%)	*p* value	*n* (%)	*p* value	*n* (%)	*p* value	*n* (%)	*p* value	*n* (%)	*p* value	Mean (SD)	*p* value
Maternal age (years)															
≤ 24	414 (8.2)	192 (46.4)	< 0.001	76 (18.4)	< 0.001	165 (39.9)	< 0.001	43 (10.4)	0.219	143 (34.5)	< 0.001	117 (28.3)	0.002	1.78 (1.42)	< 0.001
25–29	1429 (28.3)	679 (47.5)		290 (20.3)		404 (28.3)		110 (7.7)		457 (32.0)		315 (22.0)		1.58 (1.34)	
30–35	2212 (43.8)	1220 (55.2)		536 (24.2)		585 (26.5)		184 (8.3)		808 (36.5)		548 (24.8)		1.75 (1.38)	
≥ 36	1000 (19.8)	579 (57.9)		314 (31.4)		307 (30.7)		95 (9.5)		398 (39.8)		283 (28.3)		1.98 (1.42)	
Maternal parity															
Nulliparous	2634 (52.1)	1321 (50.2)	< 0.001	637 (24.2)	0.186	757 (28.7)	0.233	203 (7.7)	0.080	950 (36.1)	0.763	543 (20.6)	< 0.001	1.67 (1.35)	< 0.001
Primiparous	1524 (30.1)	851 (55.8)		346 (22.7)		425 (27.9)		142 (9.3)		533 (35.0)		426 (28.0)		1.79 (1.39)	
Multiparous	897 (17.7)	498 (55.5)		233 (26.0)		279 (31.1)		87 (9.7)		323 (36.0)		294 (32.8)		1.91 (1.46)	
Maternal BMI in the first trimester (kg/m^2^)															
< 25	3022 (59.8)	1519 (50.3)	< 0.001	543 (18.0)	< 0.001	812 (26.9)	< 0.001	231 (7.6)	< 0.001	1024 (33.9)	< 0.001	645 (21.3)	< 0.001	1.58 (1.33)	< 0.001
25–29.9	1206 (23.9)	671 (55.6)		306 (25.4)		358 (29.7)		101 (8.4)		442 (36.7)		332 (27.5)		1.83 (1.37)	
30–34.9	519 (10.3)	305 (58.8)		211 (40.7)		179 (34.5)		56 (10.8)		200 (38.5)		159 (30.6)		2.14 (1.43)	
35–39.9	203 (4.0)	114 (56.2)		100 (49.3)		73 (36.0)		26 (12.8)		88 (43.3)		81 (39.9)		2.37 (1.57)	
≥ 40	105 (2.1)	61 (58.1)		56 (53.3)		39 (37.1)		18 (17.1)		52 (49.5)		46 (43.8)		2.59 (1.55)	
Smoking															
No	4151 (82.1)	2167 (52.2)	0.169	922 (22.2)	< 0.001	1150 (27.7)	< 0.001	317 (7.6)	< 0.001	1463 (35.2)	0.176	993 (23.9)	< 0.001	1.69 (1.37)	< 0.001
Before pregnancy or passive smoking	317 (6.3)	175 (55.2)		83 (26.2)		95 (30.0)		38 (12.0)		113 (35.6)		87 (27.4)		1.86 (1.38)	
During pregnancy	587 (11.6)	328 (55.9)		211 (35.9)		216 (36.8)		77 (13.1)		230 (39.2)		183 (31.2)		2.12 (1.46)	
Daily caffeine intake in the first trimester (mg/day)^a^															
0–300	1366 (71.0)	672 (49.2)	0.593	307 (22.5)	0.117	396 (29.0)	0.923	100 (7.3)	0.984	484 (35.4)	0.515	280 (20.5)	0.017	1.64 (1.34)	0.224
> 300	558 (29.0)	282 (50.5)		144 (25.8)		163 (29.2)		41 (7.3)		189 (33.9)		142 (25.4)		1.72 (1.40)	
Physical activity during pregnancy (more than 30 min/week)															
0–2	1420 (28.1)	732 (51.5)	0.605	375 (26.4)	0.043	460 (32.4)	0.007	130 (9.2)	0.045	524 (36.9)	0.500	395 (27.8)	0.024	1.84 (1.41)	0.023
3–4	1958 (38.7)	1048 (53.5)		469 (24.0)		532 (27.2)		142 (7.3)		675 (34.5)		455 (23.2)		1.70 (1.35)	
5–6	1008 (19.9)	542 (53.8)		217 (21.5)		281 (27.9)		102 (10.1)		366 (36.3)		251 (24.9)		1.75 (1.39)	
≥ 7	669 (13.2)	348 (52.0)		155 (23.2)		188 (28.1)		58 (8.7)		241 (36.0)		162 (24.2)		1.72 (1.44)	
Marital status															
Married	2566 (50.8)	1314 (51.2)	0.018	604 (23.5)	0.333	722 (28.1)	< 0.001	197 (7.7)	0.001	890 (34.7)	0.277	618 (24.1)	0.019	1.69 (1.37)	< 0.001
Cohabitated or in a registered partnership	2264 (44.8)	1222 (54.0)		564 (24.9)		644 (28.4)		202 (8.9)		831 (36.7)		572 (25.3)		1.78 (1.41)	
Single or widow	225 (4.5)	134 (59.6)		48 (21.3)		95 (42.2)		33 (14.7)		85 (37.8)		73 (32.4)		2.08 (1.38)	
Education (years)															
≤ 12	522 (10.3)	303 (58.0)	0.094	136 (26.1)	0.156	204 (39.1)	< 0.001	63 (12.1)	0.005	209 (40.0)	0.142	166 (31.8)	< 0.001	2.07 (1.42)	< 0.001
13–15	1224 (24.2)	639 (52.2)		317 (25.9)		398 (32.5)		115 (9.4)		434 (35.5)		336 (27.5)		1.83 (1.40)	
16–18	2329 (46.1)	1216 (52.2)		536 (23.0)		592 (25.4)		183 (7.9)		829 (35.6)		536 (23.0)		1.67 (1.38)	
> 18	980 (19.4)	512 (52.2)		227 (23.2)		267 (27.2)		71 (7.2)		334 (34.1)		225 (23.0)		1.67 (1.33)	
Employment															
Employed	3981 (78.8)	2145 (53.9)	0.014	987 (24.8)	0.040	1081 (27.2)	< 0.001	310 (7.8)	< 0.001	1427 (35.8)	0.055	946 (23.8)	< 0.001	1.73 (1.38)	< 0.001
Unemployed	542 (10.7)	267 (49.3)		122 (22.5)		208 (38.4)		70 (12.9)		210 (38.7)		188 (34.7)		1.96 (1.44)	
Student	532 (10.5)	258 (48.5)		107 (20.1)		172 (32.3)		52 (9.8)		169 (31.8)		129 (24.2)		1.67 (1.41)	
Infertility treatments prior to the current pregnancy															
No	4630 (91.6)	2405 (51.9)	< 0.001	1104 (23.8)	0.247	1343 (29.0)	0.589	397 (8.6)	0.811	1633 (35.3)	0.025	1150 (24.8)	0.425	1.73 (1.39)	0.009
Yes	425 (8.4)	265 (62.4)		112 (26.4)		118 (27.8)		35 (8.2)		173 (40.7)		113 (26.6)		1.92 (1.36)	
Pregestational diabetes															
No	5019 (99.3)	2654 (52.9)	0.312	1206 (24.0)	0.600	1449 (28.9)	0.556	427 (8.5)	0.250	1792 (35.7)	0.691	1252 (24.9)	0.438	1.75 (1.39)	0.548
Yes	36 (0.7)	16 (44.4)		10 (27.8)		12 (33.3)		5 (13.9)		14 (38.9)		11 (30.6)		1.89 (1.79)	
Pregestational hypertension															
No	5006 (99.0)	2641 (52.8)	0.370	1190 (23.8)	< 0.001	1441 (28.8)	0.064	426 (8.5)	0.352	1785 (35.7)	0.295	1241 (24.8)	0.001	1.74 (1.39)	< 0.001
Yes	49 (1.0)	29 (59.2)		26 (53.1)		120 (40.8)		6 (12.2)		21 (42.9)		22 (44.9)		2.53 (1.46)	
Oral corticosteroid medication															
No	5021 (99.3)	2649 (52.8)	0.294	1209 (24.1)	0.635	1449 (28.9)	0.409	429 (8.5)	0.954	1792 (35.7)	0.506	1251 (24.9)	0.164	1.75 (1.39)	0.240
Yes	34 (0.7)	21 (61.8)		7 (20.6)		12 (35.3)		3 (8.8)		14 (41.2)		12 (35.3)		2.03 (1.47)	
Number of fetuses															
Singleton	4984 (98.6)	2629 (52.7)	0.402	1191 (23.9)	0.027	1434 (28.8)	0.088	426 (8.5)	0.977	1772 (36.6)	0.031	1248 (25.0)	0.449	1.75 (1.39)	0.041
Twins	71 (1.4)	41 (57.7)		25 (35.2)		27 (38.0)		6 (8.5)		34 (47.9)		15 (21.1)		2.08 (1.52)	
	5055 (100)	2670 (52.8)		1216 (24.1)		1461 (28.9)		432 (8.5)		1806 (35.7)		1263 (25.0)			

Abbreviation: BMI, body mass index.

^a^Data available in 1924 women.

^b^Sum of six different problematic sleep outcome variables. Each quality equals 1; minimum value 0 (no sleep disturbance during pregnancy), maximum value 6 (all six sleep disturbances during pregnancy).

Most maternal characteristics studied were significantly associated with one or more sleep disturbances. Among these, first‐trimester BMI showed the strongest association with all six types of sleep disturbances (Table 2). In contrast, higher daily caffeine intake was significantly associated only with a higher frequency of weekly daytime sleepiness, while the use of oral corticosteroids was not associated with any of the sleep variables.

Overall, sleep disturbances were more common among older women (≥ 36 years), multiparous women, those with obesity, smokers, women with the lowest levels of physical activity before pregnancy, those who were single or widowed, those who had lower educational attainment, or those who were unemployed. Furthermore, pregestational hypertension and infertility treatment prior to the current pregnancy were more prevalent among these women.

Higher maternal age, higher parity, higher first‐trimester BMI, smoking, single or widowed marital status, lower education, unemployment, low physical activity, infertility treatment, pregestational hypertension, and twin pregnancy were all predictors of a greater burden of sleep disturbances during pregnancy. Among these, first‐trimester BMI and pregestational hypertension were the strongest predictors. A linear association was observed between first‐trimester BMI and the sleep disturbance burden score: severely obese women had a mean score of 2.59 compared to 1.58 among normal‐weight women. Additionally, nulliparous women had a lower mean burden score (1.67) than primiparous (1.79) or multiparous (1.91) women. Interestingly, maternal age showed a roughly U‐shaped association with the sleep disturbance burden score: women aged 25–29 years had the lowest score (1.58), while those aged ≥ 36 years had the highest (1.98). No associations were found between the sleep disturbance burden score and caffeine intake, pregestational diabetes, or corticosteroid use.

### 3.3. GDM, Gestational Weight Gain, and Sleep Disturbances

Table 3 presents data on GDM, gestational weight gain, and their associations with sleep disturbances among 4976 women with births. GDM was diagnosed in 1151 women (23.1%). In 88.8% of these cases (1022/1151), the diagnosis was made before 28 weeks of gestation. Among those diagnosed, the majority (932/1151; 81.0%) were treated with lifestyle interventions, whereas 219 women (19.0%) received insulin and/or metformin therapy.

**Table 3:** Gestational diabetes, gestational weight gain, and poor sleep quality during the second trimester among 4976 pregnant women.

	Total	Worse sleep quality compared to nonpregnancy		Weekly snoring		Sleep duration ≤ 6 or ≥ 9 h		Often difficulties in falling asleep		Often nighttime or early morning awakenings		Weekly daytime sleepiness		Sleep disturbance burden^a^	
	*n*(%)	*n*(%)	*p*value	*n*(%)	*p*value	*n*(%)	*p*value	*n*(%)	*p*value	*n*(%)	*p*value	*n*(%)	*p*value	Mean (SD)	*p*value
Gestational diabetes mellitus (GDM)															
No	3825 (76.9)	1973 (51.6)	0.002	834 (21.8)	< 0.001	1065 (27.8)	0.007	290 (7.6)	< 0.001	1313 (34.3)	< 0.001	883 (23.1)	< 0.001	1.66 (1.36)	< 0.001
Yes	1151 (23.1)	655 (56.9)		363 (31.5)		368 (32.0)		132 (11.5)		462 (40.1)		364 (31.6)		2.04 (1.45)	
Diagnosed < 28 GWS	1022 (88.8)	588 (57.5)	0.226	330 (32.3)	0.122	323 (31.6)	0.452	117 (11.4)	0.952	411 (40.2)	0.882	324 (31.7)	0.873	2.05 (1.47)	0.450
Diagnosed ≥ 28 GWS	129 (11.2)	67 (51.9)		33 (25.6)		45 (34.9)		15 (11.6)		51 (39.5)		40 (31.0)		1.95 (1.27)	
Lifestyle treatment for GDM	932 (81.0)	517 (55.5)	0.043	265 (28.4)	< 0.001	298 (32.0)	0.998	99 (10.6)	0.063	368 (39.5)	0.350	277 (29.7)	0.004	1.96 (1.40)	< 0.001
Insulin or metformin therapy for GDM	219 (19.0)	138 (63.0)		98 (44.7)		70 (32.0)		33 (15.1)		94 (42.9)		87 (39.7)		2.37 (1.58)	
Weight gain during pregnancy (kg)^b^															
≤ 9	903 (17.9)	475 (52.6)	0.235	238 (26.4)	0.055	283 (31.3)	< 0.001	95 (10.5)	0.023	317 (35.1)	0.018	244 (27.0)	0.018	1.83 (1.45)	< 0.001
10–12	1036 (20.5)	534 (51.5)		236 (22.8)		269 (26.0)		72 (6.9)		364 (35.1)		226 (21.8)		1.64 (1.35)	
13–15	1174 (23.2)	599 (51.0)		274 (23.3)		308 (26.2)		98 (8.3)		402 (34.3)		281 (23.9)		1.67 (1.40)	
16–19	1051 (20.8)	568 (54.0)		237 (22.5)		304 (28.9)		77 (7.3)		371 (35.3)		266 (25.3)		1.73 (1.34)	
≥ 20	532 (10.5)	300 (56.4)		148 (27.8)		187 (35.2)		53 (10.0)		226 (42.5)		152 (28.6)		2.00 (1.37)	
Weight gain during pregnancy excluding women with GDM (kg)^c^															
≤ 9	561 (15.5)	285 (50.8)	0.071	116 (20.7)	0.072	171 (30.5)	< 0.001	50 (8.9)	0.427	188 (33.5)	0.033	132 (23.5)	0.047	1.68 (1.40)	< 0.001
10–12	795 (22.0)	388 (48.8)		176 (22.1)		197 (24.8)		53 (6.7)		261 (32.8)		162 (20.4)		1.56 (1.33)	
13–15	968 (26.8)	483 (49.9)		200 (20.7)		243 (25.1)		74 (7.6)		324 (33.5)		209 (21.6)		1.69 (1.35)	
16–19	872 (24.1)	471 (54.0)		186 (21.3)		245 (28.1)		59 (6.8)		302 (34.6)		210 (24.1)		1.68 (1.32)	
≥ 20	418 (11.6)	233 (55.7)		114 (27.3)		147 (35.2)		36 (8.6)		173 (41.4)		115 (27.5)		1.96 (1.38)	
Weight gain during pregnancy including only women with GDM (kg)^d^															
≤ 9	342 (31.6)	190 (55.6)	0.679	122 (35.7)	0.034	112 (32.7)	0.884	45 (13.2)	0.220	129 (37.7)	0.422	112 (32.7)	0.384	2.08 (1.50)	0.467
10–12	241 (22.3)	146 (60.6)		60 (24.9)		72 (29.9)		19 (7.9)		103 (42.7)		64 (26.6)		1.93 (1.37)	
13–15	206 (19.0)	116 (56.3)		74 (35.9)		65 (31.6)		24 (11.7)		79 (38.3)		72 (35.0)		2.09 (1.52)	
16–19	179 (16.5)	97 (54.2)		51 (28.5)		59 (33.0)		18 (10.1)		69 (38.5)		56 (31.3)		1.96 (1.38)	
≥ 20	114 (10.5)	67 (58.8)		34 (29.8)		40 (35.1)		17 (14.9)		53 (46.5)		37 (32.5)		2.18 (1.33)	

Abbreviations: GDM, gestational diabetes mellitus; GWS, gestational weeks.

^a^Sum of six different problematic sleep outcome variables. Each quality equals 1; minimum value 0 (no sleep disturbance during pregnancy), maximum value 6 (all six sleep disturbances during pregnancy).

^b^Data available in 4696 women.

^c^Data available in 3614 women.

^d^Data available in 1082 women.

Women diagnosed with GDM exhibited a significantly greater variety of sleep disturbances compared to those without GDM. Among women with GDM, those receiving pharmacological treatment reported worse sleep quality, more weekly snoring, more difficulties in falling asleep, and daytime sleepiness than those treated with lifestyle counseling alone.

Women with GDM gained less weight during pregnancy compared to those without GDM (mean 12.2 kg, SD 6.2 vs. mean 14.1 kg, SD 4.9; *p* < 0.001). Gestational weight gain was not associated with sleep disturbances among women with GDM (Table 3). However, among women without GDM, weight gain was associated with most of the six evaluated sleep disturbances. Notably, these associations often followed a U‐shaped pattern, with women who gained ≥ 20 kg during pregnancy experiencing the highest prevalence of sleep disturbances. The differences were significant between groups when comparing women who gained ≥ 20kg to those who gained less for all other sleep problems except difficulties in falling asleep. Similar associations were not detected between excessive weight gain and any sleep problems among women who had GDM (*p* values between 0.136 and 0.819).

### 3.4. Sleep Disturbances, Covariates, and GDM

Table 4 presents both unadjusted and adjusted analyses of the association between sleep disturbances and GDM. In the univariate analysis, all sleep disturbances were associated with an increased risk of GDM compared to women without the respective disturbances. The highest odds were observed for snoring ≥ 3 nights per week (OR 1.90; 95% CI 1.58–2.29) and daily daytime sleepiness (OR 1.95; 95% CI 1.49–2.54).

**Table 4:** Multivariable analysis of sleep quality in relation to gestational diabetes among 4976 pregnant women.

	Gestational diabetes			
	*n*/*N*(%)	OR (95% CI)	aOR (95% CI)^a^	aOR (95% CI)^b^
Sleep quality compared to nonpregnancy				
No change or better	496/2348 (21.1)	1	1	1
Worse	655/2628 (24.9)	1.24 (1.09–1.42)	1.11 (0.96–1.28)	1.13 (0.98–1.31)
*p* value		0.002	0.150	0.097
Snoring				
No	788/3779 (20.9)	1	1	1
Yes, 1–2 nights a week	159/586 (27.1)	1.41 (1.16–1.72)	1.07 (0.87–1.33)	1.08 (0.87–1.35)
Yes, ≥ 3 nights a week	204/611 (33.4)	1.90 (1.58–2.29)	1.11 (0.91–1.37)	1.13 (0.92–1.40)
*p* value		< 0.001	0.536	0.461
Sleep duration (h)				
7–8	783/3543 (22.1)	1	1	1
≤ 6	154/543 (28.4)	1.40 (1.14–1.71)	1.20 (0.96–1.49)	1.20 (0.96–1.50)
≥ 9	214/890 (24.0)	1.12 (0.94–1.33)	1.04 (0.87–1.26)	1.05 (0.87–1.27)
*p* value		0.004	0.265	0.277
Difficulties in falling asleep				
No	521/2391 (21.8)	1	1	1
Sometimes	498/2163 (23.0)	1.07 (0.93–1.23)	0.95 (0.81–1.10)	0.97 (0.83–1.13)
Often or almost every night	132/422 (31.3)	1.63 (1.30–2.05)	1.35 (1.05–1.72)	1.37 (1.06–1.77)
*p* value		< 0.001	0.019	0.026
Nighttime or early morning awakenings				
No or sometimes	689/3201 (21.5)	1	1	1
Often or almost every night	462/1775 (26.0)	1.28 (1.12–1.47)	1.16 (1.00–1.34)	1.14 (0.98–1.33)
*p* value		< 0.001	0.050	0.085
Daytime sleepiness				
No or cannot define	787/3729 (21.1)	1	1	1
Frequently, at least weekly	274/984 (27.8)	1.44 (1.23–1.69)	1.21 (1.02–1.44)	1.24 (1.04–1.48)
Almost daily	90/263 (34.2)	1.95 (1.49–2.54)	1.52 (1.14–2.03)	1.55 (1.15–2.10)
*p* value		< 0.001	0.004	0.002
Sleep disturbance burden				
0, none	179/1038 (17.2)	1	1	1
1	276/1371 (20.1)	1.21 (0.98–1.49)	1.07 (0.86–1.33)	1.08 (0.86–1.36)
2	291/1200 (24.3)	1.54 (1.25–1.89)	1.27 (1.02–1.58)	1.28 (1.02–1.61)
3	219/770 (28.4)	1.91 (1.52–2.39)	1.37 (1.08–1.74)	1.40 (1.09–1.79)
≥ 4	186/597 (31.2)	2.17 (1.71–2.75)	1.41 (1.09–1.82)	1.46 (1.12–1.90)
*p* value		< 0.001	0.012	0.010

Abbreviations: aOR, adjusted odds ratio; CI, confidence interval; OR, odds ratio.

^a^Adjusted for maternal age, body mass index, smoking, and physical training; *N* = 4976.

^b^Adjusted for maternal age, body mass index, smoking, physical training, and gestational weight gain; *N* = 4696.

After adjusting for maternal age, BMI, smoking, and physical activity in the multivariable analysis, difficulties in falling asleep occurring often or almost every night (aOR 1.35; 95% CI 1.05–1.72), nighttime or early morning awakenings occurring often (aOR 1.16; 95% CI 1.00–1.34), and frequent or daily daytime sleepiness (aOR 1.21; 95% CI 1.02–1.44 and aOR 1.52; 95% CI 1.14–2.03, respectively) remained significantly associated with GDM. When gestational weight gain was further included in the analysis, the associations remained for frequent or daily daytime sleepiness (aOR 1.24; 95% CI 1.04–1.48 and aOR 1.55; 95% CI 1.15–2.10, respectively) and difficulties in falling asleep (aOR 1.37; 95% CI 1.06–1.77). Sleep duration and snoring were not associated with GDM after adjustments.

A sensitivity analysis restricted to women who underwent OGTT (*n* = 3765) yielded similar results (data not shown).

## 4. Discussion

Based on a retrospective study involving nearly 5000 Finnish pregnant women, an increase in the variety of sleep problems experienced during the second trimester was found to be associated with a higher frequency of GDM. Associations were even stronger among women who required pharmacological treatment for gestational diabetes compared with those who were managed with lifestyle measures. In the univariate analysis, the risk of GDM was associated with a subjectively reported decline in sleep quality compared with the prepregnancy period, weekly snoring, shorter sleep duration, frequent difficulty falling asleep, recurrent nighttime or early morning awakenings, and weekly episodes of daytime sleepiness. Among these factors, the strongest association was observed between difficulty falling asleep and GDM. Our findings highlight that several different sleep problems are very common among Finnish pregnant women, particularly among those with GDM, pregestational hypertension, and/or maternal overweight or obesity. Since these maternal characteristics frequently coexist, we aimed to evaluate their independent associations through multivariable adjustments.

A meta‐analysis conducted by Zhang et al. [14], involving 16 studies and 2,551,017 pregnant women, identified various sleep disturbances that were associated with an increased risk of GDM. However, many earlier studies and studies included in this analysis were limited by small sample sizes or a low prevalence of women with GDM and/or focused on only one specific sleep issue (e.g., sleep duration or snoring) [14, 20]. However, there are also prior studies that have examined the relationship between multiple sleep variables and GDM simultaneously [15, 16, 19, 27–30]. These studies commonly utilized the Pittsburgh Sleep Quality Index (PSQI), which includes several questions assessing sleep quality and provides a cumulative score categorizing sleep as either good or poor. All but one of these studies reported a significant correlation between poor sleep quality and the risk of GDM [28].

Our findings are consistent with these previous results, demonstrating that a greater number of sleep disturbances is associated with an increased risk of GDM. However, our study extends previous literature by demonstrating that the association between sleep problems and the risk of GDM rather accumulates across a variety of sleep issues. Additionally, our study showed that women diagnosed with GDM and treated with metformin or insulin reported a greater variety of sleep disturbances than women who received only lifestyle counseling. This suggests that a greater variety of sleep disturbances may be associated with higher blood glucose levels. Chen et al. [16] demonstrated a linear relationship between declining sleep quality and elevated blood glucose levels, as well as an increased risk of GDM, in a longitudinal study of 4550 Chinese women. Furthermore, sleep disturbances have been linked to the development of type 2 diabetes in nonpregnant populations in many previous studies [12, 31]. In a recent postpartum follow‐up study by Yin et al. [32] among women with a history of GDM, those with shorter sleep duration and snoring had the highest risk of developing type 2 diabetes after pregnancy. More frequent snoring was also associated with adverse cardiometabolic markers, including higher levels of HbA1c, C‐peptide, and insulin [32]. Thus, recognizing and managing sleep disturbances during and after pregnancy is also important for the mother’s long‐term metabolic health.

Poor sleep quality measured using the PSQI has also been associated with increased levels of insulin resistance among pregnant women [33]. However, the biological mechanisms underlying these associations remain poorly understood. Epidemiological studies suggest that sleep deprivation may impair glucose tolerance, increase insulin resistance, and contribute to pancreatic *β*‐cell dysfunction, ultimately leading to GDM [34]. One proposed mechanism involves elevated oxidative stress, which is associated with impaired glucose metabolism and insulin resistance [34, 35]. Additionally, sleep restriction may increase circulating levels of inflammatory mediators such as C‐reactive protein, interleukin‐6, and tumor necrosis factor‐*α*, all of which are implicated in the pathogenesis of insulin resistance [35, 36]. Another potential mechanism is the overactivation of the sympathetic nervous system, which may lead to increased hepatic glucose production and reduced insulin sensitivity [34, 35]. Furthermore, dysregulation of the hypothalamic–pituitary–adrenal (HPA) axis may result in elevated secretion of growth hormone and cortisol, both of which contribute to insulin resistance and impaired glucose tolerance [34, 35]. Sleep disturbances may also affect the regulation of appetite and satiety hormones, such as leptin and ghrelin, potentially leading to weight gain and further insulin resistance [34, 35]. This is reinforced by our finding that excessive gestational weight gain was significantly associated with sleep problems in pregnant women who did not have GDM.

Our study has several strengths. To our knowledge, it is the first to examine multiple sleep disturbances independently during the second trimester of pregnancy and their association with the risk of GDM. We observed a high prevalence of GDM (23.1%), and more than 40% of the study population was overweight or obese, reflecting national trends in Finland [3]. Furthermore, we prospectively collected a wide range of maternal characteristics, allowing for comprehensive adjustment for potential confounding variables.

Our study also has several limitations. First, we used a self‐designed sleep questionnaire that was not standardized, and its reliability and validity have not been formally assessed. Our sleep‐related questions were included as part of a broader late‐pregnancy questionnaire in the large KuBiCo cohort, which also covered a wide range of other health‐related exposures alongside sleep. Second, sleep patterns may fluctuate throughout pregnancy, but we assessed sleep disturbances only during the second trimester. Third, the questionnaire did not cover all possible sleep‐related issues, such as daytime napping or restless legs syndrome.

Furthermore, we lacked information on whether participants had preexisting sleep disturbances before pregnancy, and psychiatric factors that can influence sleep quality, such as depression and anxiety, were not included in the analysis. Both maternal depression and the use of psychotropic medications have been shown to increase the risk of developing GDM and are also closely associated with overweight and obesity [37–39]. However, 52.8% of all participants reported a deterioration in sleep quality during pregnancy compared to the prepregnancy period, indicating that worsening sleep is a very common phenomenon even in normal pregnancy.

Lastly, due to the retrospective design of the study, recall bias or stress following the GDM diagnosis, which may affect maternal responses, cannot be ruled out. However, the prevalence of different sleep problems was similar among women diagnosed with GDM before the 28th week and those diagnosed thereafter. Furthermore, gestational weight gain among women with GDM, which partly correlates with adherence to the GDM diet and treatment, was not associated with sleep problems, even among women whose weight gain was excessive during pregnancy. Thus, we believe that recalling sleep problems earlier in pregnancy was not substantially affected by recall bias or stress due to GDM diagnosis.

## 5. Conclusions

We found a significant and independent association between various sleep disturbances and the risk of GDM, and this association became stronger with an increasing number of different sleep disturbances experienced during pregnancy. These findings suggest that sleep disturbances should be given greater attention in prenatal care and may warrant consideration for routine screening in the first trimester, as well as possible interventions during pregnancy in the most severe cases. We suggest that improved sleep education and support during pregnancy might help women adopt healthier lifestyle habits, contributing to more optimal gestational weight gain and healthier pregnancy and long‐term health overall.

## Author Contributions

L.K.N., S.M.L. and S.H. designed the KuBiCo study. L.K.N. contributed to the acquisition of KuBiCo data for this study. S.H. was responsible for the nutrition work package of KuBiCo. M.T., L.K.N., and S.M.L. supervised the conduct of this study. L.K.N. and J.L. contributed to the statistical analysis. J.L. and J.M. wrote the manuscript under the supervision of L.K.N. and S.M.L. All authors have reviewed and commented on the manuscript and approved the final version.

## Funding

Funding was provided by the University of Eastern Finland, Kuopio University Hospital, and the National Institute of Health and Welfare. Open access publishing facilitated by Ita‐Suomen yliopisto, as part of the Wiley ‐ FinELib agreement.

## Disclosure

All authors critically reviewed the drafts and approved the final manuscript.

## Ethics Statement

The Research Ethics Committee of the Hospital District of Central Finland in Jyväskylä, Finland, reviewed and approved the KuBiCo study on November 15, 2011 (Number 18U/2011). Electronic informed written consent was obtained from the mothers at the time of recruitment during pregnancy.

## Conflicts of Interest

The authors declare no conflicts of interest.

## Data Availability

The data that support the findings of this study are available on request from the corresponding author. The data are not publicly available due to privacy or ethical restrictions.

## References

[1] World Health Organization , Definition, Diagnosis and Classification of Diabetes Mellitus and Its Complications: Report of a WHO Consultation. Part 1, Diagnosis and Classification of Diabetes Mellitus World Health Organization, 2026, Available from:[Internet]. 1999 [cited https://apps.who.int/iris/handle/10665/66040.

[2] International Diabetes Federation , The Diabetes Atlas 10th Edition, 2021, International Diabetes Federation, [Internet]. 2021 [cited 2025 Jan 25]. Available from: IDF Diabetes Atlas | Global Diabetes Data & Statistics.

[3] Finnish Medical Society Duodecim Gestational Diabetes , Finnish Current Care Guidelines, 2025, Working Group Set Up by the Finnish Medical Society Duodecim and the Finnish Diabetes Association and the Finnish Society of Obstetrics and Gynecology, Helsinki: [Internet]. 2024 [cited Available from: http://www.kaypahoito.fi/.

[4] McIntyre H. D. , Catalano P. , Zhang C. , Desoye G. , Mathiesen E. R. , and Damm P. , Gestational Diabetes Mellitus, Nature Reviews Disease Primers. (2019) 5, no. 1, 10.1038/s41572-019-0098-8, 2-s2.0-85068918533. 31296866

[5] Farrar D. , Simmonds M. , Bryant M. , Sheldon T. A. , Tuffnell D. , Golder S. , Dunne F. , and Lawlor D. A. , Hyperglycaemia and Risk of Adverse Perinatal Outcomes: Systematic Review and Meta-Analysis, BMJ. (2016) 354, i4694, 10.1136/bmj.i4694, 2-s2.0-84988310728. 27624087 PMC5021824

[6] Metzger B. E. , Lowe L. P. , Dyer Alan R. , Trimble E. R. , Chaovarindr U. , Coustan D. R. et al., Hyperglycemia and Adverse Pregnancy Outcomes, New England Journal of Medicine. (2008) 358, no. 19, 1991–2002, 10.1056/NEJMoa0707943, 2-s2.0-43249096940, 18463375. 18463375

[7] Committee on Practice Bulletins—Obstetrics , ACOG Practice Bulletin: Gestational Diabetes Mellitus, 2025, Committee on Practice Bulletins—Obstetrics, Available from: https://pubmed.ncbi.nlm.nih.gov/29370047/.

[8] Pengo M. F. , Won C. H. , and Bourjeily G. , Sleep in Women Across the Life Span, Chest. (2018) 154, no. 1, 196–206, 10.1016/j.chest.2018.04.005, 2-s2.0-85048978255, 29679598. 29679598 PMC6045782

[9] Mindell J. A. , Cook R. A. , and Nikolovski J. , Sleep Patterns and Sleep Disturbances Across Pregnancy, Sleep Medicine. (2015) 16, no. 4, 483–488, 10.1016/j.sleep.2014.12.006, 2-s2.0-84926517202, 25666847. 25666847

[10] Lu Q. , Zhang X. , Wang Y. , Li J. , Xu Y. , Song X. , Su S. , Zhu X. , Vitiello M. V. , Shi J. , Bao Y. , and Lu L. , Sleep Disturbances During Pregnancy and Adverse Maternal and Fetal Outcomes: A Systematic Review and Meta-Analysis, Sleep Medicine Reviews. (2021) 58, 101436, 10.1016/j.smrv.2021.101436, 33571887. 33571887

[11] Antza C. , Kostopoulos G. , Mostafa S. , Nirantharakumar K. , and Tahrani A. , The Links Between Sleep Duration, Obesity and Type 2 Diabetes Mellitus, J Endocrinol.(2021) 252, no. 2, 125–141, 10.1530/JOE-21-0155, 34779405. 34779405 PMC8679843

[12] Cappuccio F. P. , Strazzullo P. , and Miller M. A. , Quantity and Quality of Sleep and Incidence of Type 2 Diabetes: A Systematic Review and Meta-Analysis, Diabetes Care. (2010) 33, no. 2, 414–420, 10.2337/dc09-1124, 2-s2.0-75149137910, 19910503. 19910503 PMC2809295

[13] Holliday E. G. , Magee C. A. , Kritharides L. , Banks E. , and Attia J. , Short Sleep Duration Is Associated With Risk of Future Diabetes but Not Cardiovascular Disease: A Prospective Study and Meta-Analysis, PLoS One. (2013) 8, no. 11, 82305, 10.1371/journal.pone.0082305, 2-s2.0-84896728853. PMC3840027 24282622

[14] Zhang X. , Zhang R. , Cheng L. , Wang Y. , Ding X. , Fu J. , Dang J. , Moore J. , and Li R. , The Effect of Sleep Impairment on Gestational Diabetes Mellitus: A Systematic Review and Meta-Analysis of Cohort Studies, Sleep Medicine. (2020) 74, 267–277, 10.1016/j.sleep.2020.05.014, 32862011. 32862011

[15] Zhong C. , Chen R. , Zhou X. , Xu S. , Li Q. , Cui W. , Wang W. , Li X. , Wu J. , Liu C. , Xiao M. , Sun G. , Yang X. , Hao L. , and Yang N. , Poor Sleep During Early Pregnancy Increases Subsequent Risk of Gestational Diabetes Mellitus, Sleep Medicine. (2018) 46, 20–25, 10.1016/j.sleep.2018.02.014, 2-s2.0-85045714929, 29773207. 29773207

[16] Chen H. , He Y. , Zeng X. , Chen Q. , Zhou N. , Yang H. , Zhou W. , Zhang L. , Yang R. , Huang Q. , and Zhang H. , Sleep Quality Is an Independent Predictor of Blood Glucose and Gestational Diabetes Mellitus: A Longitudinal Study of 4550 Chinese Women, Nature and Science of Sleep. (2022) 14, 609–620, 10.2147/NSS.S353742, 35431589. PMC9012300 35431589

[17] Wang H. , Leng J. , Li W. , Wang L. , Zhang C. , Li W. , Liu H. , Zhang S. , Chan J. , Hu G. , Yu Z. , and Yang X. , Sleep Duration and Quality, and Risk of Gestational Diabetes Mellitus in Pregnant Chinese Women, Diabetic Medicine. (2017) 34, no. 1, 44–50, 10.1111/dme.13155, 2-s2.0-84973279369, 27154471. 27154471

[18] Myoga M. , Tsuji M. , Tanaka R. , Shibata E. , Askew D. J. , Aiko Y. , Senju A. , Kawamoto T. , Hachisuga T. , Araki S. , and Kusuhara K. , Impact of Sleep Duration During Pregnancy on the Risk of Gestational Diabetes in the Japan Environmental and Children’s Study (JECS), BMC Pregnancy Childbirth. (2019) 19, no. 1, 10.1186/s12884-019-2632-9, 31818260. PMC6902452 31818260

[19] Wang W. , Meng H. , Liu Y. , Yin W. , Li Z. , Wan M. , Zou L. , and Zhang D. , Effects of Sleep Duration and Sleep Quality in Early Pregnancy and Their Interaction on Gestational Diabetes Mellitus, Sleep Breath. (2022) 26, no. 1, 489–496. 33929688 10.1007/s11325-021-02391-3

[20] Rawal S. , Hinkle S. N. , Zhu Y. , Albert P. S. , and Zhang C. , A Longitudinal Study of Sleep Duration in Pregnancy and Subsequent Risk of Gestational Diabetes: Findings From a Prospective, Multiracial Cohort, American Journal of Obstetrics and Gynecology. (2017) 216, no. 4, 399.e1–399.e8, 10.1016/j.ajog.2016.11.1051, 2-s2.0-85016237387. PMC6331053 27939328

[21] KuBiCo-Kuopio Birth Cohort–Kuopion Syntymäkohorttitutkimus. [Internet]. 2024 [cited 2026, Jan 25]. Available from: Kuopion syntymäkohorttitutkimus (KuBiCo) - UEFConnect.

[22] Huuskonen P. , Keski-Nisula L. , Heinonen S. , Voutilainen S. , Tuomainen T. P. , Pekkanen J. , Lampi J. , Lehto S. M. , Haaparanta H. , Elomaa A. P. , Voutilainen R. , Backman K. , Kokki H. , Kumpulainen K. , Paananen J. , Vähäkangas K. , and Pasanen M. , Kuopio Birth Cohort-Design of a Finnish Joint Research Effort for Identification of Environmental and Lifestyle Risk Factors for the Wellbeing of the Mother and the Newborn Child, BMC Pregnancy Childbirth. (2018) 18, no. 1, 10.1186/s12884-018-2013-9, 2-s2.0-85053599410, 30241516. PMC6150990 30241516

[23] Kukkonen A. , Hantunen S. , Voutilainen A. , Ruusunen A. , Uusitalo L. , Backman K. , Voutilainen R. , Pasanen M. , Kirjavainen P. V. , and Keski-Nisula L. , Maternal Caffeine, Coffee and Cola Drink Intake and the Risk of Gestational Diabetes–Kuopio Birth Cohort, Prim Care Diabetes.(2024) 18, no. 3, 362–367, 10.1016/j.pcd.2024.02.005, 38423827. 38423827

[24] Zhang C. , Rawal S. , and Chong Y. S. , Risk Factors for Gestational Diabetes: Is Prevention Possible?, Diabetologia. (2016) 59, no. 7, 1385–1390, 10.1007/s00125-016-3979-3, 2-s2.0-84966605800, 27165093. 27165093 PMC6364673

[25] Viswanathan M. , Siega-Riz A. M. , Moos M. K. , Deierlein A. , Mumford S. , Knaack J. et al., Outcomes of Maternal Weight Gain, Evidence Report/Technology Assessment. (2008) 168, 1–223. PMC4781425 18620471

[26] Hedderson M. M. , Gunderson E. P. , and Ferrara A. , Gestational Weight Gain and Risk of Gestational Diabetes Mellitus, Obstetrics and Gynecology. (2010) 115, no. 3, 597–604, 10.1097/AOG.0b013e3181cfce4f, 2-s2.0-77649147669. 20177292 PMC3180899

[27] Ferrari U. , Künzel H. , Tröndle K. , Rottenkolber M. , Kohn D. , Fugmann M. , Banning F. , Weise M. , Sacco V. , Hasbargen U. , Hutter S. , Parhofer K. G. , Kloiber S. , Ising M. , Seissler J. , and Lechner A. , Poor Sleep Quality Is Associated With Impaired Glucose Tolerance in Women After Gestational Diabetes, Journal of Psychiatric Research. (2015) 65, 166–171, 10.1016/j.jpsychires.2015.02.012, 2-s2.0-84929503005, 25930074. 25930074

[28] Sharma S. K. , Nehra A. , Sinha S. , Soneja M. , Sunesh K. , Sreenivas V. , and Vedita D. , Sleep Disorders in Pregnancy and Their Association With Pregnancy Outcomes: A Prospective Observational Study, Sleep Breath. (2016) 20, no. 1, 87–93, 10.1007/s11325-015-1188-9, 2-s2.0-84961164396, 25957617. 25957617

[29] Cai S. , Tan S. , Gluckman P. D. , Godfrey K. M. , Saw S. M. , Teoh O. H. , Chong Y. S. , Meaney M. J. , Kramer M. S. , Gooley J. J. , and on behalf of the GUSTO study group , Sleep Quality and Nocturnal Sleep Duration in Pregnancy and Risk of Gestational Diabetes Mellitus, Sleep. (2017) 40, no. 2, 10.1093/sleep/zsw058, 2-s2.0-85014113975. 28364489

[30] Peivandi S. , Habibi A. , Hosseini S. H. , Khademloo M. , Raisian M. , and Pournorouz H. , Evaluation of Sleep Quality and Duration in Pregnancy and Risk of Gestational Diabetes Mellitus: A Prospective Follow-Up Study, Biomedicine. (2021) 11, no. 2, 24–29, 10.37796/2211-8039.1139, 35223401. 35223401 PMC8824243

[31] Medic G. , Wille M. , and Hemels M. E. , Short- and Long-Term Health Consequences of Sleep Disruption, Nature And Science Of Sleep. (2017) 9, no. 9, 151–161, 10.2147/NSS.S134864, 2-s2.0-85030661946. PMC5449130 28579842

[32] Yin X. , Bao W. , Ley S. H. , Yang J. , Cuffe S. B. , Yu G. , Chavarro J. E. , Liu P. , Zhou J. H. , Tobias D. K. , Hu F. B. , and Zhang C. , Sleep Characteristics and Long-Term Risk of Type 2 Diabetes Among Women With Gestational Diabetes, AMA Network Open. (2025) 8, no. 3, e250142, 10.1001/jamanetworkopen.2025.0142, 40042841. PMC11883505 40042841

[33] Du M. , Liu J. , Han N. , Zhao Z. , Luo S. , and Wang H. , Exploring the Mediating Role of Serum Retinol-Binding Protein 4 in the Relationship Between Sleep Quality and Insulin Resistance in Pregnant Women, Diabetes Research and Clinical Practice. (2021) 176, 108866, 10.1016/j.diabres.2021.108866, 34023339. 34023339

[34] Briançon-Marjollet A. , Weiszenstein M. , Henri M. , Thomas A. , Godin-Ribuot D. , and Polak J. , The Impact of Sleep Disorders on Glucose metabolism: endocrine and molecular mechanisms, Diabetology & metabolic syndrome. (2015) 7, no. 1, 10.1186/s13098-015-0018-3, 2-s2.0-84926361751, 25834642. PMC4381534 25834642

[35] Izci-Balserak B. and Pien G. W. , The Relationship and Potential Mechanistic Pathways Between Sleep Disturbances and Maternal Hyperglycemia, Current Diabetes Reports. (2014) 14, no. 2, 10.1007/s11892-013-0459-8, 2-s2.0-84895076679, 24398662. PMC4065785 24398662

[36] Irwin M. R. , Olmstead R. , and Carroll J. E. , Sleep Disturbance, Sleep Duration, and Inflammation: A Systematic Review and Meta-Analysis of Cohort Studies and Experimental Sleep Deprivation, Biological Psychiatry. (2016) 80, no. 1, 40–52, 10.1016/j.biopsych.2015.05.014, 2-s2.0-84933544583, 26140821. 26140821 PMC4666828

[37] Gong H. and Zhao Y. , Association Between Body Roundness Index and Sleep Disorder: The Mediating Role of Depression, BMC Psychiatry. (2025) 25, no. 1, 10.1186/s12888-025-06664-z, 40055626. PMC11889924 40055626

[38] Ruohomäki A. , Toffol E. , Upadhyaya S. , Keski-Nisula L. , Pekkanen J. , Lampi J. , Voutilainen S. , Tuomainen T. P. , Heinonen S. , Kumpulainen K. , Pasanen M. , and Lehto S. M. , The Association Between Gestational Diabetes Mellitus and Postpartum Depressive Symptomatology: A Prospective Cohort Study, Journal of Affective Disorders. (2018) 241, 263–268, 10.1016/j.jad.2018.08.070, 2-s2.0-85051663934, 30138811. 30138811

[39] Kananen A. , Bernhardsen G. P. , Lehto S. M. , Huuskonen P. , Kokki H. , and Keski-Nisula L. , Quetiapine and Other Antipsychotic Medications During Pregnancy: A 15-Year Follow-Up of a University Hospital Birth Register, Nordic Journal of Psychiatry. (2023) 77, no. 7, 651–660, 10.1080/08039488.2023.2205852, 37149788. 37149788

